# Objective and owner-reported outcomes after modified cranial closing wedge ostectomy: a case series

**DOI:** 10.1007/s11259-023-10261-4

**Published:** 2023-11-18

**Authors:** Jenny M. Kovacs, Parisa Mazdarani, Michelle B. M. Nielsen, James E. Miles

**Affiliations:** 1 Lunds Djursjukhus Evidensia, Porfyrvägen 6, Lund, 22478 Sweden; 2 College of Veterinary Medicine, University of Florida, 2015 SW 16th Ave, Gainesville, FL 32608 USA; 3 Department of Veterinary Clinical Sciences, University Hospital for Companion Animals, University of Copenhagen, Dyrlægevej 16, Frederiksberg C, 1870 Denmark

**Keywords:** Cranial closing wedge ostectomy, Cranial cruciate ligament, Dog, Complications, Gait analysis

## Abstract

Immediate and longer-term outcomes of a cranial closing wedge ostectomy variant for management of canine cranial cruciate ligament disease were assessed in this single-center retrospective consecutive study. Records and radiographs were retrieved and assessed by three independent observers to evaluate tibial plateau angle, anatomical-mechanical axis angle, tibial tuberosity distalization, and mechanical axis length before and after surgery. Kinetic gait analysis and owner questionnaires were used to assess clinical outcomes. Seventeen stifles from fifteen dogs were evaluated radiographically. Mean error from target tibial plateau angle was 0.4 degrees. Anatomical-mechanical axis angles reduced from mean 2.9 degrees preoperatively to mean − 0.9 degrees postoperatively. Tibial tuberosity distalization was mean 5.0% of mechanical axis length, and mean reduction in mechanical axis length was 0.1%. Increased tibial plateau angles were noted in 8/17 stifles, with a mean of 9.6 degrees at short-term follow-up. Major complications were observed in 9/17 stifles. Long term follow-up (mean 832 days) was obtained with gait analysis in 8/15 dogs and with questionnaire in 11/15. Most dogs (9/11) were weakly to moderately affected by osteoarthritis symptoms. All values for peak vertical force and vertical impulse normalized to body weight exceeded local lower reference limits for normal dogs, indicating acceptable limb use. Satisfactory immediate and long-term clinical outcomes appear to be possible with this technique, but the high incidence of shorter-term complications may caution against the technique or the fixation and management described here.

## Introduction

Cranial closing wedge ostectomy (CCWO) is a long-established tibial osteotomy technique for stabilization of the cranial cruciate ligament (CCL) deficient stifle (Slocum and Devine 1984). During surgery, a wedge of bone is removed from the proximal tibia, and the proximal fragment rotated about the wedge apex to reduce the tibial plateau angle (TPA) before rigid internal fixation. Several clinical case series using this technique or variations upon it have been reported (Bailey et al. 2007; Campbell et al. 2016; Christ et al. 2018; Corr and Brown 2007; Duerr et al. 2008; Frederick and Cross 2017; Guénégo et al. 2021; Kuan et al. 2009; Macias et al. 2002; Oxley et al. 2013; Selmi and Filho 2001; Terreros and Daye 2020). Variations include different methods of calculating the wedge size, positioning of the wedge apex, and reduction criteria (cranial or caudal cortical alignment).

A recent review found that variants with more proximally positioned wedges tended to yield smaller errors in achieving target TPA (Miles and Nielsen 2023). Only limited data relevant to previous concerns about tibial tuberosity distalization, associated patella baja, and tibial shortening following CCWO raised by Corr and Brown (2007) could be found. A recent clinical and *in silico* study found larger TPA errors with the isosceles wedge technique than historical data would suggest across a heterogeneous population including excessive TPA (Banks et al. 2023). Another *in silico* study of a modified CCWO and a neutral wedge technique (Story et al. 2023) showed that rather than tibial shortening, lengthening may actually occur with these procedures, while another indicated only limited changes are likely across several techniques (Miles et al. 2023). Historical complication rates for CCWO range from 0 to 32%, with major complications - as defined by Cook et al. (2010) – seen in up to 24% of cases (Miles and Nielsen 2023).

Center of rotation of angulation (CORA) principles may be used to stabilize CCL deficient stifles (Raske et al. 2013). This approach treats the proximal tibia as if affected by an angular deformity, defining proximal and distal anatomical axes, and allowing calculation of the necessary correction angle and placement of the angular correction axis (ACA) to achieve accurate realignment of the bone. We hypothesized that a variant CCWO using these principles (C-CCWO) could reliably achieve target TPA while minimally affecting mechanical axis (MA) length and achieving reduction of the anatomical-mechanical axis angle (AMA), as well as achieve acceptable long-term clinical and functional outcomes.

## Methods

Institutional ethical approval was obtained for this study (2021-33). This was a single-center, retrospective, consecutive case series for radiographic analysis of dogs managed with C-CCWO coupled with prospective measurement of gait characteristics. Cases were operated at the corresponding author’s institution between November 2017 and November 2019, with long-term follow-up during October and November 2021. This case series is reported in line with the PROCESS 2020 guidelines (Agha et al. 2020).

### Perioperative management

Patients were premedicated with methadone (0.3 mg/kg) and a sedative before induction of anesthesia with propofol and maintenance with isoflurane in oxygen. An epidural containing bupivacaine (0.5 mg/kg) and morphine (0.1 mg/kg) blended to a volume of 1ml/4.5 kg body mass (maximum 6 ml) was given preoperatively. Intraarticular morphine (0.2 mg/kg) was administered following joint exploration. Postoperative analgesia included transdermal fentanyl (2–3 µg/kg) for 3 days, with a non-steroidal anti-inflammatory drug at recommended doses and/or paracetamol (10–15 mg/kg 2–3 times daily) for at least 1–2 weeks. Perioperative antibiosis consisted of cefazoline (20 mg/kg) administered at induction and every 90 min during surgery.

### Surgical planning and technique

**Fig. 1:**
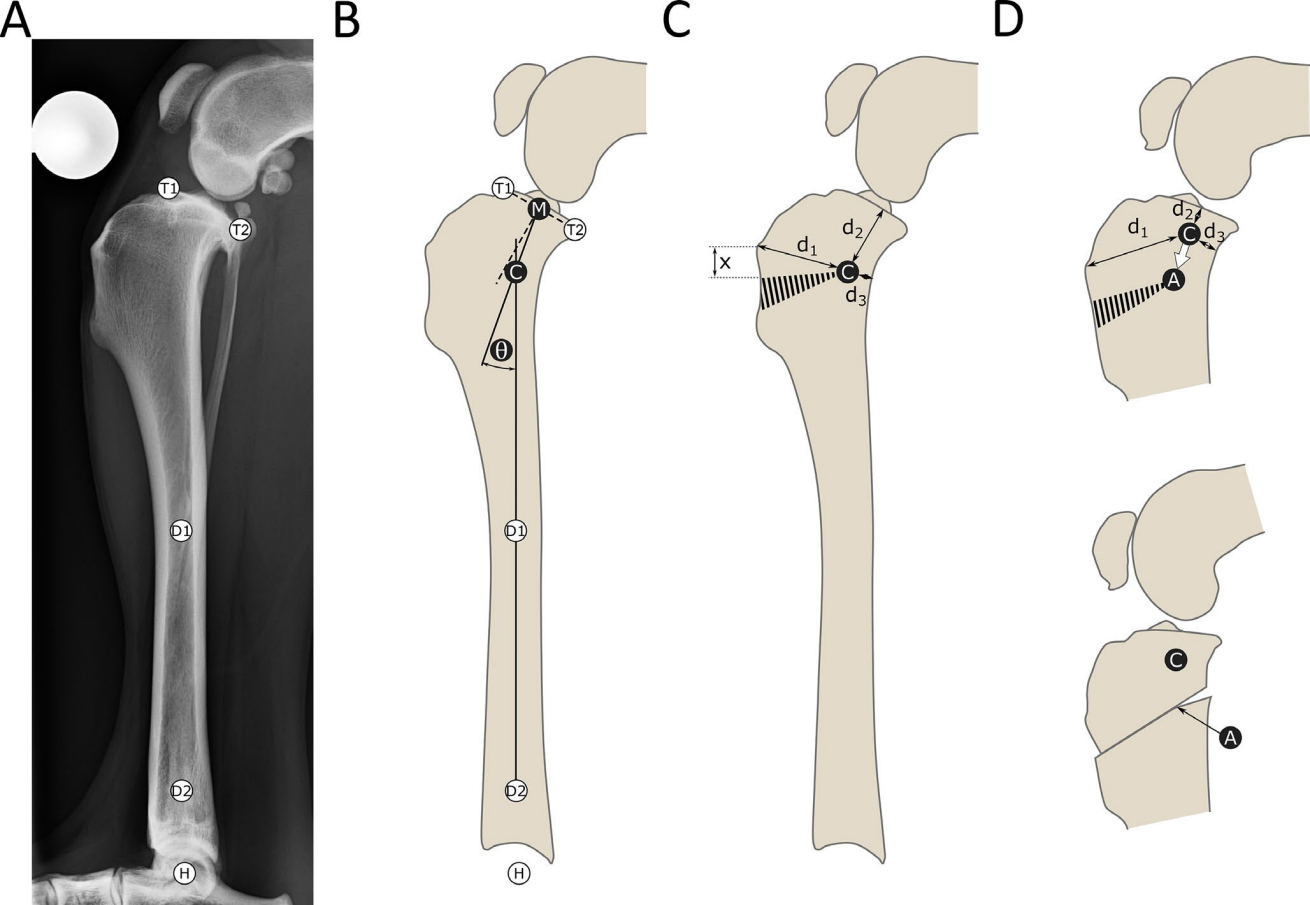
Planning for and execution of CORA-based closing wedge tibial ostectomy procedure. **A** - Five landmarks were identified on a full-length tibial radiograph: T1, T2 – cranial and caudal aspects of the tibial plateau; D1, D2 – diaphyseal centers at 50% of tibial length and just proximal to the distal tibial flare, H – center of the hock joint. **B** – landmarks T1 and T2 defined the tibial plateau, and the proximal anatomical axis was drawn as a line oriented 5° caudal to the perpendicular to the tibial plateau, passing through its midpoint (M). Landmarks D1 and D2 defined the distal anatomical axis. The intersection of the two axes defined the center of rotation of angulation (CORA) point (C), and the angle (θ) between them the wedge angle to achieve a tibial plateau angle of 5°. **C** – during surgery, distances d1, d2 and d3 were used to transfer the location of the CORA point to the patient. This location defined the proposed angular correction axis (ACA) and was adjusted distally if necessary to accommodate implants proximally. The ACA became the wedge apex, with the base on the cranial border of the tibia, and the proximal and distal cuts separated by angle θ (striped region). The proximal cut was positioned 5–10 mm distal to the tibial tuberosity (distance x). The proximal cut was continued through to the caudal cortex to separate the proximal and distal fragments. **D** – Intraoperative correction of ACA positioning in another dog. Ideally the CORA and ACA locations should be the same for a neutral wedge osteotomy. If the proposed CORA (C) defined by distances d1, d2 and d3 was too proximal for placement of the selected implants, the ACA (A) was repositioned more distally. Placement of the wedge apex at the ACA and reduction of the cranial aspect of the ostectomy results in a rotation about the ACA

Before surgery, the placement and size of the wedge ostectomy was calculated according to CORA principles (Fig. 1) (Paley 2002). Planning was performed using mediolateral full tibial radiographs including the hock joint, centered over the stifle joint, with the stifle and hock joints flexed to 90°, and with a maximum deviation from full femoral condylar superimposition of < 2 mm (Fettig et al. 2003; Reif and Probst 2003).The distal anatomical axis was defined by a line passing through diaphyseal midpoints at 50% of tibial length and just proximal to the distal tibial flare (Miles 2020). The proximal anatomical axis was defined by a line bisecting the tibial plateau and angled 5° caudal to a perpendicular to the tibial plateau, in order to achieve a target TPA of 5° once the proximal and distal tibial axes were aligned during surgery. The intersection of these axes marked the CORA location, and the acute angle between them the wedge angle for the target TPA of 5°. The CORA location was mapped on radiographs using distances from the tibial tuberosity, joint line, and the caudal cortex as references for surgery (distances d1, d2, and d3 in Fig. 1C).

Following arthroscopy and/or arthrotomy to confirm CCL deficiency and evaluate the menisci, a standard medial approach to the proximal tibia was made. The CORA was localized using preoperative measurements by triangulation using a surgical ruler to measure the distance d1 from the tibial tuberosity, d2 from a 23G needle placed at the level of the joint through the medial collateral ligament, and d3 from the caudal aspect of the tibia (Fig. 1C), and represented the ideal location of the ACA for a neutral wedge osteotomy (Paley 2002). Distal adjustment was made as needed to permit placement of implants proximally (Fig. 1D), and a 2 mm hole was drilled at the selected point and designated as the operative ACA. An osteometer and saw guide designed for the triple tibial osteotomy procedure^1^ was inserted into this hole and used to position the two osteotomies accurately to remove a wedge equal to the wedge angle. The proximal osteotomy was continued through the ACA to the caudal cortex. After removing the wedge, the ostectomy gap was reduced using compression forceps and temporarily stabilized with a pin, using the 2 mm drill hole to guide alignment of the proximal and distal fragments in a craniocaudal direction. Internal fixation was achieved using a plate in combination with secondary stabilization, such as an additional smaller plate, or pin and tension band. Postoperative radiographs were obtained, and patients discharged the following day once appropriate analgesia was ensured. Strict rest was advised. A follow-up examination was performed 10–14 days after surgery and radiographic examinations scheduled for 5–8 weeks postoperatively prior to a controlled return to normal exercise. Rehabilitation and physiotherapy were routinely recommended.

### Data extraction and radiographic assessment

Medical records from dogs with CCL deficiency in which C-CCWO had been performed at our institution were reviewed and assessed for patient age, breed, sex, fixation methods, and incidence and type of post-surgical complications, using published definitions (Cook et al. 2010).

**Fig. 2:**
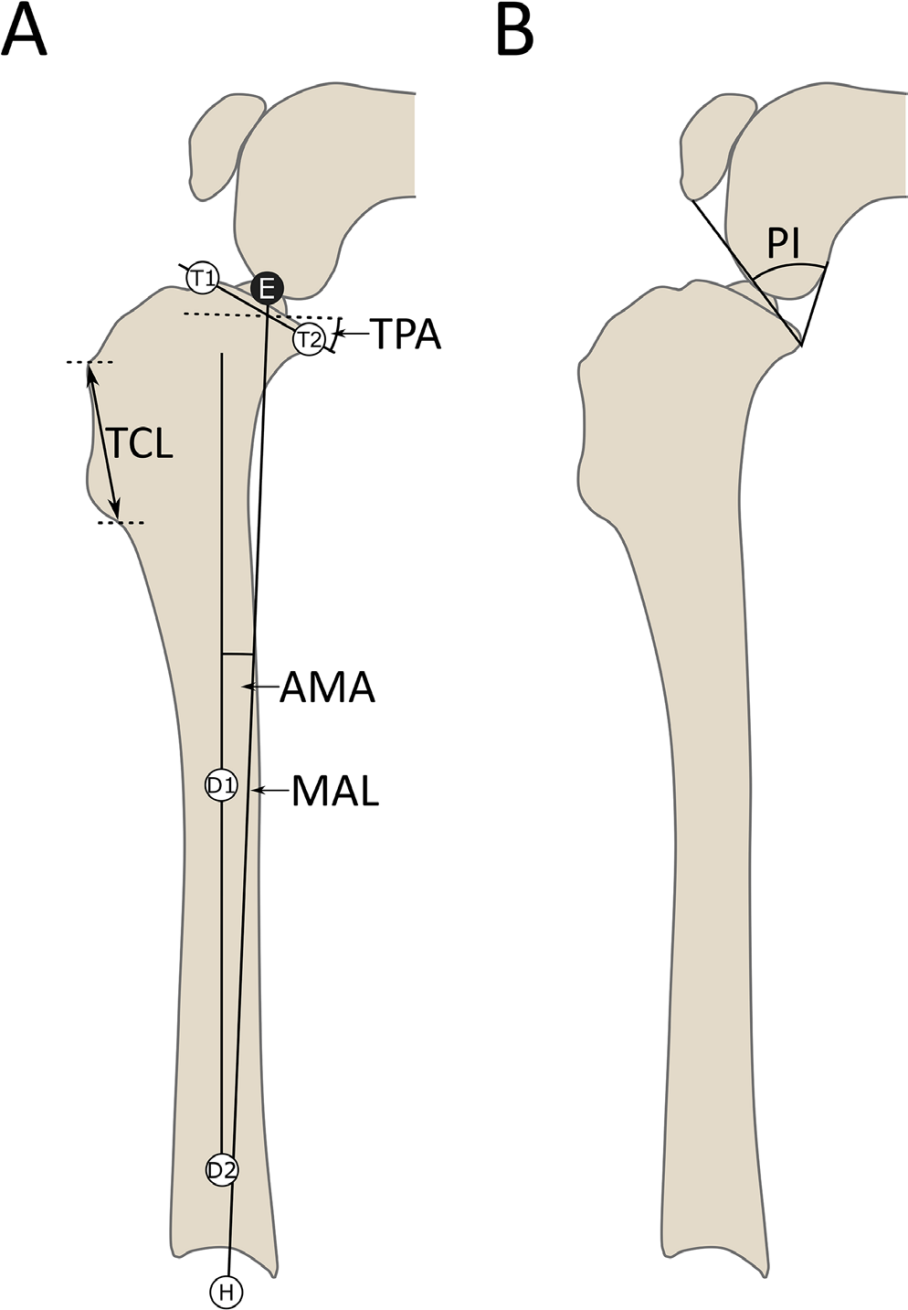
Measurement protocol for angular measurements. The same landmarks as in Fig. 1 were utilised along with the center of the tibial eminence (E). **A** –The tibial plateau angle (TPA) was found as the angle between the line connecting T1 and T2 and the perpendicular to the mechanical axis defined by points E and H. The distance between E and H defined the mechanical axis length (MAL). Points D1 and D2 defined the distal anatomical axis, and the angle between this and the mechanical axis was the anatomical-mechanical axis angle (AMA). The tibial crest length (TCL) was measured between the cranial insertion of the patellar ligament and the distal aspect of the crest. **B** – measurement of the patellar index (Allberg and Miles 2020), using the caudal aspects of the femoral and tibial condyles, and the distal pole of the patella to determine the acute angle (PI)

Digital preoperative, postoperative and short-term follow-up radiographs were retrieved for each patient. If post-operative or short-term follow-up radiographs did not include the entire distal tibia and the hock joint, freely available software^2^ was used to make composite images from the preoperative tibia as previously described (Hazenfield et al. 2014; Miles et al. 2012). Three experienced surgeons working independently identified the CORA location and correction angle from the preoperative radiographs (Fig. 1) and measured TPA and AMA at each time point (Fig. 2). Tibial crest length, MA length, and patellar index (Allberg and Miles 2020) were measured on pre- and postoperative radiographs (Fig. 2). The patellar index was the acute angle between lines connecting the caudal aspects of the femoral and tibial condyles, and the distal pole of the patellar and caudal tibial condyles. TPA was measured using the cranial and caudal aspects of the tibial plateau and the MA, passing through the centers of the tibiotarsal joint and the tibial eminence. AMA was measured between this mechanical axis and the distal anatomical axis, defined above. Tibial crest length was measured from the distal insertion of the patellar ligament to the distal end of the cranial border of the tibia, and MA length from the tibial eminence to the center of the tibiotarsal joint.

**Fig. 3:**
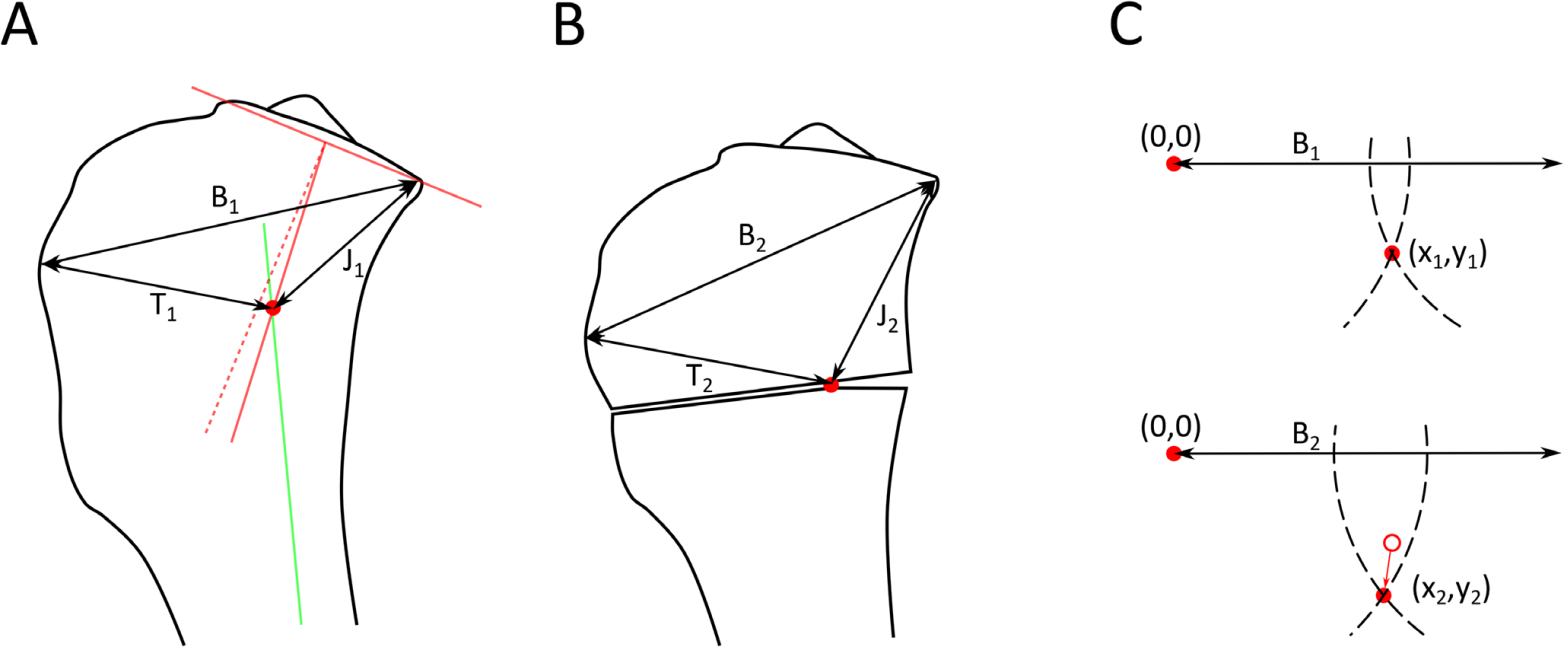
Measurement of center of rotation of angulation (CORA) and angular correction axis (ACA) locations. **A** - Using the method shown in Fig. 1, the CORA location (circle) was identified on each preoperative radiograph and 3 linear measurements used to define its two-dimensional position: B_1_ – baseline from tibial tuberosity to caudal joint surface, T_1_ – distance from tibial tuberosity to CORA, J_1_ – distance from caudal joint surface to CORA. **B** – On each postoperative radiograph, the ACA was identified using the remnants of the 2 mm drill hole used for the osteotomy guide, and linear measurements B_2_, T_2_ and J_2_ found. **C** – By placing the origin (0,0) at the tibial tuberosity and defining the baseline (B1, B2) as the x-axis, the coordinates of the CORA (x_1_, y_1_) and ACA (x_2_, y_2_) could be derived from the intersections of two circles with radii T_1_ and J_1_ or T_2_ and J_2_, and the distance between these coordinates found as the square root of ((x_1_-x_2_)^2^+(y_1_-y_2_)^2^)

One surgeon identified the ACA location on the postoperative radiographs for comparison with the calculated CORA location (Fig. 3). The distance between the tibial tuberosity and proximal joint surface point was measured and transferred from the preoperative to the postoperative radiographs. With this distance acting as a baseline the CORA and ACA locations could be triangulated and the distance between them calculated.

### Veterinary and owner assessment of long-term function

Owners were invited to bring their dogs for long-term follow-up: if this was declined, they were requested to complete a translation of the Liverpool Osteoarthritis in Dogs (LOAD) questionnaire^3^ during a telephone interview. Signed consent was obtained from each owner, and clinical and orthopedic examinations performed on each dog before kinetic analysis using a pressure-sensitive walkway (Tekscan Medical #3140) and proprietary software (Tekscan Walkway 7.66). Dogs were walked on a loose leash by an experienced handler in one direction for 5 acceptable recordings. Recordings were considered acceptable if all four limbs contacted the mat, forward velocity was 0.9–1.1 m/s, acceleration was ± 0.5 m/s^2^, and no overt head movements (looking to either side, or up to the handler) were observed. Body-weight normalized peak vertical force (PVF_%BW_) and vertical impulse (VI_%BW_), averaged across the 5 recordings, were retrieved for each dog. Values were compared to pooled reference intervals for hind limbs from healthy dogs given in Table 1 of Nielsen et al. (2020). Attending owners completed a LOAD questionnaire.

### Statistical analysis

Statistical analysis was performed using commercial software (SPSS 27.0 for Windows, IBM Corp, Armonk, NY). Data were assessed for normality using the Shapiro-Wilk test and quartile-quartile plots, and homoscedasticity using Koenker’s test and graphical evaluation.

Postoperative and short-term follow-up values for TPA and AMA were compared using paired sample t-tests. Statistical significance was set at 5%. Interobserver agreement for TPA and AMA measurements at each time point, and correction angle, was assessed using the within-subject standard deviation and repeatability coefficient (Bland and Altman 1996). Results for PVF_%BW_ and VI_%BW_ were visualized graphically.

## Results

### Demographic data

In total, 17 stifle joints from 15 dogs were included (Table 1). Of these, 4 were Labrador retrievers and the remaining dogs mainly large breeds; only two dogs weighed less than 10 kg. Nine out of 15 dogs were female. Mean body mass was 31.6 kg (SD 14.9; range 7.9–66.0 kg) and the mean age at initial surgery was 80 months (SD 31 months; range 8 months – 134 months).

**Table 1:** Demographic data. For dogs operated bilaterally, mass shown is the average at time of surgery for each limb

Dog ID	Limb	Breed	Sex	Mass (kg)	Age (months)
1	L	Labrador retriever	F	28.0	112
2	L	Labrador retriever	M	40.8	95
2	R	Labrador retriever	-	-	99
3	L	German Shepherd dog	F	31.5	76
4	L	Labrador retriever	M	37.0	76
5	L	Rottweiler	F	35.0	86
6	R	Mixed breed	M	13.5	91
7	L	Great Dane	F	64.3	49
7	R	Great Dane	-	-	51
8	R	Cavalier King Charles spaniel	F	9.2	106
9	L	Labrador retriever	M	42.0	75
10	L	Alaskan Malamute	M	35.0	42
11	R	Mixed breed	F	44.0	53
12	L	Golden retriever	F	26.0	8
13	L	Jack Russell terrier	F	7.9	106
14	R	Australian Shepherd dog	F	20.0	134
15	L	Golden retriever	M	40.5	99

### Observer agreement

Based on overlap of confidence intervals, there were no significant differences between interobserver TPA and AMA measurements from preoperative, postoperative and short-term follow-up radiographs (Table 2). The coefficient of repeatability for TPA based on preoperative values was 3.5° (95% CI: 2.7°; 4.4°) and was used to define a minimum clinically detectable difference for identifying the rock-back phenomenon.

**Table 2:** Interobserver reliability of radiographic measurements. Values are point estimates of the within-subject standard deviation with 95% confidence intervals in parentheses for images obtained preoperatively, postoperatively and at short-term follow-up

	Preoperative	Postoperative	Follow-up
TPA (°)	1.3 (0.9; 1.6)	1.9 (1.5; 2.4)	2.0 (1.5; 2.5)
AMA (°)	0.4 (0.3; 0.6)	0.4 (0.3; 0.6)	0.5 (0.4; 0.6)
Wedge (°)	1.4 (1.0; 1.7)	-	-
TTD (mm)	-	1.7 (1.3; 2.1)	-
MALC (mm)	-	1.0 (0.8; 1.3)	-

TPA – tibial plateau angle; AMA – anatomical-mechanical axis angle; Wedge – calculated wedge angle; TTD – tibial tuberosity distalization (preoperative to postoperative); MALC – mechanical axis length change (preoperative to postoperative)

### Clinical measurements

Mean preoperative TPA was 23.5° (SD 2.9°; range 18.4° – 27.1°), reduced to 5.4° (SD 2.3°; range 0.8° − 11.9°) postoperatively, giving a mean error from target of 0.4° (SD 2.3°; range − 4.2° – 6.9°). Mean TPA increased to 9.6° (SD 3.9°; range 3.3° − 20.0°) on short-term follow-up radiographs (*P* < 0.001, Cohen’s d = 1.0) and using the coefficient of repeatability as the cut-off criterion, rock-back phenomenon was seen in 8/17 stifles. Mean AMA pre- and immediately postoperatively were 2.1° (SD 0.9°; range 0.8° − 3.6°) and − 0.9° (SD 0.9°; range − 2.2° – 0.7°), respectively. On short-term follow-up radiographs, AMA had increased to -0.2° (SD 1.2°; range − 2.0° − 2.9°) (*P* = 0.02, Cohen’s d = 0.7).

The mean wedge angle was 20.7° (SD 3.6°; range 14.4° – 25.9°) based on a target TPA of 5°. The mean linear difference between mapped CORA and executed ACA placement was 9 mm (SD 5 mm, range 3–22 mm). There was no clear relationship between this and patient size, and values ranged from 2 to 11% of initial MA length.

Nine out of 17 joints had a medial meniscal lesion identified at initial surgery, of which 7/9 were managed by partial meniscectomy, and 2/9 with mild edge fibrillation were managed conservatively. Two joints with normal menisci at initial surgery later developed a clinically significant meniscal lesion (Table 3).

**Table 3:** Implants, meniscal status, complications and their management in this case series

Dog ID	Limb	Joint findings	Primary fixation	Supplementary fixation	Complication	Management	Rock back?
1	L	MCHD	3.5 mm SOP TPLO-B	3.5 mm SOP 3 H	Tibial tuberosity fracture	3 H plate replaced with PTB	Y
2	L	MBHT	3.5 mm SOP TPLO	PTB	SSI	AB, implant removal	Y
2	R	MCHD	3.5 mm SOP TPLO-B	3.5 mm SOP 3 H	SSI	AB	Y
3	L	MCHD	3.5 mm SOP TPLO	PTB			
4	L	MCHD	3.5 mm SOP TPLO	PTB	Screw breakage	Implant removal and replacement	Y
5	L	MBHT	3.5 mm SOP TPLO-B	PTB			
6	R	-	2.7 mm SOP TPLO	2.0 mm SOP 3 H	Fibular fracture	None required	Y
7	L	-	3.5 mm SOP TPLO-B	PTB	Suspected SSI	AB	
7	R	-	3.5 mm SOP TPLO-B	PTB	Suspected SSI	AB	
8	R	-	2.7 mm SOP TPLO	PTB			
9	L	-	3.5 mm SOP TPLO	3.5 mm SOP 6 H + HC	LMI	Partial meniscectomy	Y
10	L	-	3.5 mm SOP TPLO	3.5 mm SOP 3 H			
11	R	-	3.5 mm SOP TPLO-B	PTB	LMI, pin loosening	Partial meniscectomy, pin removal	
12	L	-	3.5 mm Clover Leaf T-plate				Y
13	L	MCHD	2.0 mm SOP 6 H	PTB	Screw breakage, fibular fracture	Implant removal and replacement	Y
14	R	MCHF	3.5 mm SOP TPLO	2.7 mm SOP 3 H			
15	L	MCHF	3.5 mm SOP TPLO-B	3.5 mm SOP 3 H			

L, R – left, right; MCHD – medial caudal horn detachment; MBHT – medial bucket handle tear; MCHF – medial caudal horn fibrillation; SOP TPLO – String of Pearls TPLO plate (-B – broad format); SOP – String of Pearls plate (H – holes); PTB – pin and tension band; HC – hemicerclage; SSI – surgical site infection; LMI – late meniscal injury; AB – antibiotics; Rock back if change in tibial plateau angle exceeded coefficient of repeatability for measurement

Tibial tuberosity distalization, as estimated from shortening of the cranial border of the tibia, was 9.2 mm (SD 3.4 mm; range 5.3–17.6 mm) corresponding to 5.0% (SD 1.3%; range 2.9–7.4%) of preoperative MA length. Mean preoperative MA length was 183 mm (SD 45 mm; range 91–271 mm). Postoperatively, MA length was 0.2 mm (SD 2.2 mm; range − 2.7–4.9 mm) shorter, corresponding to 0.1% (SD 1.2%; range − 1.4–2.8%) of preoperative MA length.

### Complications

Major complications, requiring either medical or surgical management, were observed in 9/17 limbs (Table 3). Two cases experienced implant failure requiring removal and replacement of implants: two additional cases had implants removed due to either loosening or infection. There was no clear association between primary and supplementary fixation for each limb and complications, including rock back phenomenon as defined above.

### Long-term follow-up

Mean interval from initial operation to long-term follow-up (i.e., ignoring subsequent management of late meniscal injury or contralateral CCL rupture) was 832 days (SD 78 days; range 705–922 days). Owners of 11/15 dogs completed LOAD questionnaires, and 8 dogs were physically presented (Table 4). Two dogs had reportedly been euthanized for reasons unrelated to CCL disease or surgery and were not included in long-term follow-up analyses, and two were lost to long-term follow-up. Mean TPA for the eight dogs undergoing gait analysis was 8.3° (SD 3.2°; range 3.3° − 13.7°). Of these, 5/8 had experienced major complications, and 3/8 had no complications.

**Table 4:** Data for dogs physically presented for long-term follow-up. Time indicates the interval between the cranial closing wedge ostectomy and presentation for orthopedic examination and gait analysis – subsequent presentation for bilateral disease or management of meniscal injury were ignored when calculating this period. Tibial plateau angle (TPA) represents the TPA established on short-term radiographic follow-up evaluation 5–8 weeks postoperatively

Dog ID	Limb	Time (days)	Mass (kg)	TPA (°)	LOAD score	PVF_%BW_	VI_%BW_	Contralateral disease
1	L	705	28	12.6	6	33.1	9.5	
2	L	827	40.8	13.7	18	33.3	10.2	Operated bilaterally
2	R	-	-	9.7	-	36.1	11.1	
3	L	922	31.5	6.5	13	29.1	10.5	
4	L	864	37	9.4	2	32.7	10.1	
7	L	-	64.3	5.3	18	30.2	13.2	Operated bilaterally
7	R	889	-	7.2	-	32	13.5	
11	R	892	44	6.3	8	32	12.6	
14	R	742	20	3.3	10	44.7	12.4	Coxofemoral pain
15	L	748	40.5	9.4	22	47.3	15.3	Suspected cruciate disease

LOAD – Liverpool Osteoarthritis in Dogs; PVF_%BW_ – bodyweight-normalized peak vertical force; VI_%BW_ – bodyweight-normalized vertical impulse

The LOAD scores ranged from 2 to 22: 5/11 dogs were graded weakly affected (scores ≤ 10), 4/11 dogs were graded moderately affected (scores 11–20), and 2/11 dogs were graded severely affected (scores > 20) by articular conditions. On clinical examination, slight crepitus, mildly decreased range of motion or mild evidence of pain and/or effusion were noted in 3/9 operated joints.

**Fig. 4:**
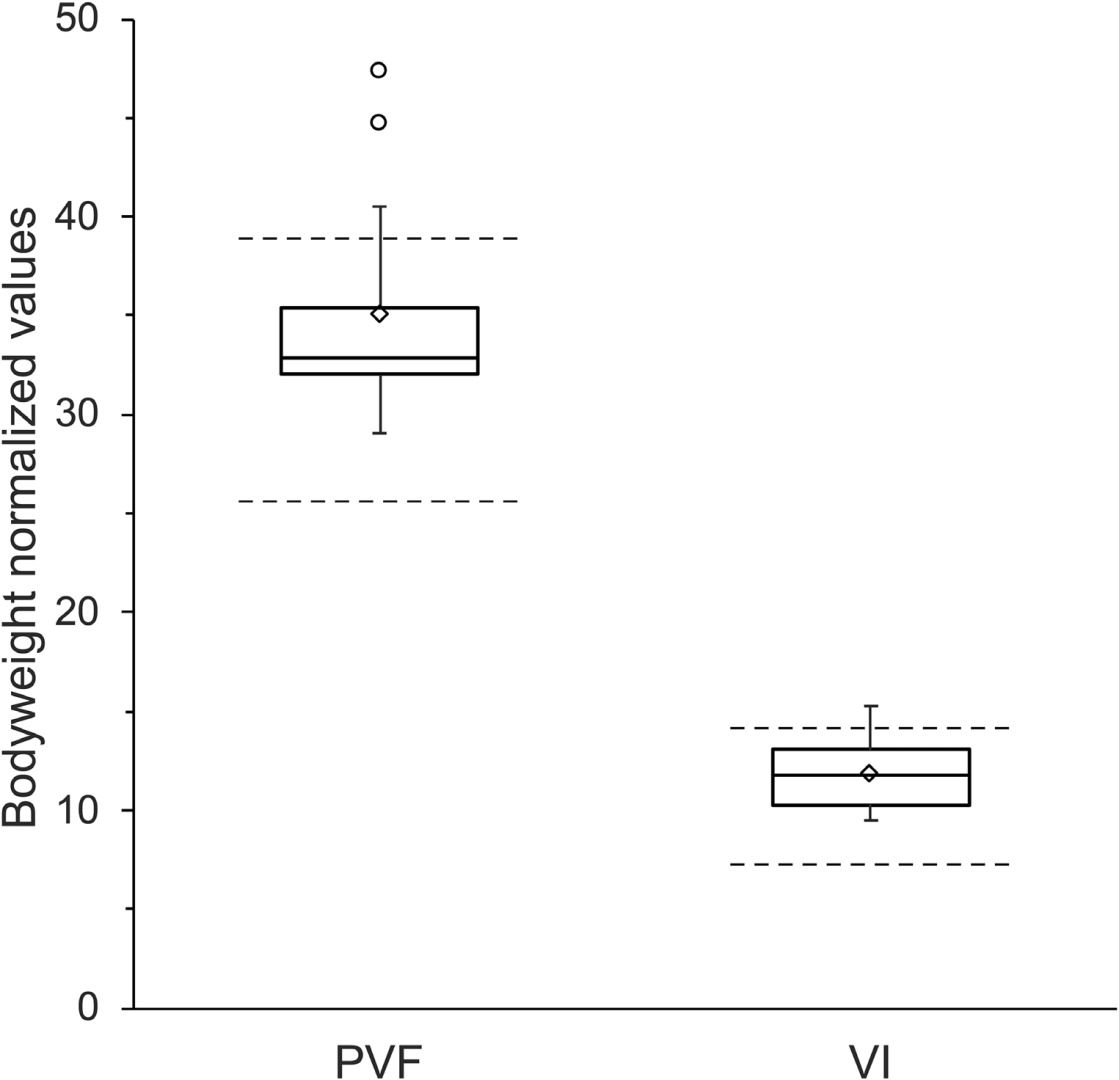
Bodyweight normalized peak vertical force and vertical impulse at long-term follow-up. Values obtained from 8 dogs (10 limbs) are shown as box and whisker plots. Boxes represent the 2nd and 3rd quartiles, with the median shown as a horizontal line and the mean as a diamond. Whiskers extend to the minima/maxima or a maximum of 1.5 times the interquartile range: outliers are represented by circles. Reference intervals derived from Nielsen et al. (2020) are shown as dashed lines

All values for PVF_%BW_ and VI_%BW_ (Fig. 4) exceeded the lower reference limit for normal dogs. Two dogs in which abnormalities of the contralateral limb were observed (suspected CCL disease and hip osteoarthritis) had PVF_%BW_ and VI_%BW_ values which exceeded the upper reference range. Spearman’s rank correlations between LOAD scores and either PVF_%BW_ or VI_%BW_ were 0.24 (*P* = 0.6) and 0.70 (*P* = 0.06), respectively.

## Discussion

The C-CCWO produced acceptable immediate postoperative TPA values with limited error from target. Complication rates exceeded those reported historically, and the incidence of rock back with associated increases in TPA was unacceptable. Despite this, long-term function in a subgroup of these patients appeared within normal limits based on kinetic gait analysis. Our hypotheses were therefore partially met.

Error in immediate postoperative TPA from target for C-CCWO was similar to overall values for modified CCWO of -0.6° and AMA-based CCWO of 0.5° (Guénégo et al. 2021; Miles and Nielsen 2023), which is unsurprising given the similarities in wedge position of these variants. Variability in postoperative TPA, as assessed by SD values, was greater than that reported for modified CCWO (Christ et al. 2018), isosceles CCWO (Oxley et al. 2013) and AMA-based CCWO (Guénégo et al. 2021) which all achieved an SD < 2°, but is consistent with values reported for tibial plateau levelling osteotomy of 1.4° to 3.7° (Coletti et al. 2014; Guénégo et al. 2021; Pagès et al. 2022; Robinson et al. 2006) and superior to a recent report of isosceles CCWO (Banks et al. 2023). We therefore consider C-CCWO to be capable of acceptable correction of TPA.

Overall complication rates were much higher than the 15.2 − 22.7% reported for tibial plateau levelling osteotomy (Beer et al. 2018; Husi et al. 2023; Knebel et al. 2020; Knight and Danielski 2018) and the 0 − 32% reported for other CCWO variants (Campbell et al. 2016; Christ et al. 2018; Frederick and Cross 2017; Kuan et al. 2009; Macias et al. 2002; Oxley et al. 2013; Terreros and Daye 2020). In addition, most of our complications were categorized as major, whereas in most other reports, there has been a large proportion of minor complications. Incidence of surgical site infection at 2–4/17 limbs markedly exceeded a recent report (Husi et al. 2023) but did not appear to contribute to implant failure in this case series. Reasons for surgical site infection are multifactorial, and while we adhered to good surgical practice in double gloving, barrier draping, intradermal suturing, and use of perioperative antibiosis (Stine et al. 2018), re-evaluation and improvement of perioperative patient management at our institution appears warranted. We did not use antibiotics postoperatively, and evidence for this practice remains controversial (Budsberg et al. 2021).

The high incidence of rock back with associated increase in TPA was disappointing and surprising given the use of locking implants and supplementary fixation in almost all cases. Despite this, most dogs had PVF_%BW_ and VI_%BW_ values within our institution’s reference range, consistent with reported findings following tibial plateau levelling osteotomy with TPA between 0° and 14° (Robinson et al. 2006). The cause of rock back may be due to inadequate sizing of the implants, surgeon error, poor postoperative control of activity, or some combination of these factors. Use of SOP TPLO plates for tibial plateau levelling has been associated with a slightly higher rate of complications but similar short-term changes in TPA compared to other locking systems (McGregor et al. 2019). The greater change in TPA from postoperative to short-term follow-up in this case series may be due to the implants also having to counteract quadriceps tension, and the supplementary fixation used being inadequate to this task. Use of compression plates and double plating, as described for AMA-based CCWO (Guénégo et al. 2021), might yield improved short-term results.

While iatrogenic patellar baja is a potential concern after CCWO, a radiographic study of cases operated with an AMA-based CCWO technique found no cases of baja, although patellar position did alter slightly (Guénégo et al. 2021). Interpretation of changes in patellar position is complicated by the alterations in stifle joint conformation and limb alignment following CCWO. We did not specifically report patellar proximodistal position, as cranial tibial subluxation in the preoperative radiographs prevented reliable comparison with the postoperative situation when using the Allberg-Miles index (Allberg and Miles 2020). In contrast, a study of the modified Maquet procedure successfully employed this index to identify patella baja (Giansetto et al. 2023). However, planning radiographs for the modified Maquet procedure typically use an extended stifle mediolateral view (Etchepareborde et al. 2011) which will tend to reduce cranial tibial subluxation compared to the positioning used in our cases.

The discrepancy measured between planned CORA and actual ACA did not noticeably affect the immediate postoperative TPA values. A geometric analysis of CORA-based levelling osteotomy showed that errors of 10 mm in ACA placement from the true CORA produced limited effects on simulated postoperative TPA (Mazdarani et al. 2021). Based on data presented in that study, maximum errors in postoperative TPA of < 2° could be expected in our population.

Use of owner-completed questionnaires is popular in canine orthopaedics due to their low cost and ease of use. In common with a larger study comparing LOAD to kinetic analyses (Walton et al. 2013), we saw only weak to moderate correlations between LOAD scores and either PVF_%BW_ or VI_%BW_. While kinetic analysis is generally considered the reference standard for evaluation of surgical outcomes, clinical cases are seldom affected by disease of only one joint or limb, particularly in older populations. This can create problems discerning differences between similarly affected hind limbs with kinetic analysis (Nielsen et al. 2020), while a more holistic approach via a questionnaire may reveal abnormalities. Clinicians using such questionnaires in translated forms (as here) should be aware of the need for construct validation, as cultural differences or framing of questions could result in results deviating from those expected in the original language version.

This study is limited by its largely retrospective nature and small numbers, particularly for the long-term follow-up, and the heterogeneous population. The long-term gait analysis is encouraging with regards to the potential for recovery despite complications and is a novel contribution to the literature around CCWO with the exception of an experimental study (Lee et al. 2007).

Satisfactory immediate and long-term clinical outcomes appear to be possible with C-CCWO. The high incidence of short- to medium-term complications may caution against its use, or the fixation and management systems used in these patients.

## Footnotes

1 Veterinary Instrumentation, Sheffield, UK, https://veterinary-instrumentation.co.uk/.

2 GIMP 2.10, The GIMP Development Team, 2021, https://www.gimp.org.

3 Elanco Animal Health, https://orthoassist.elanco.com/.
